# The Role of Macrophages in the Host’s Defense against *Sporothrix schenckii*

**DOI:** 10.3390/pathogens10070905

**Published:** 2021-07-18

**Authors:** Estela Ruiz-Baca, Armando Pérez-Torres, Yolanda Romo-Lozano, Daniel Cervantes-García, Carlos A. Alba-Fierro, Javier Ventura-Juárez, Conchita Torriello

**Affiliations:** 1 Facultad de Ciencias Químicas, Universidad Juárez del Estado de Durango, Av. Veterinaria S/N, Durango 34120, Mexico; eruiz@ujed.mx (E.R.-B.); carlos.alba@ujed.mx (C.A.A.-F.); 2 Facultad de Medicina, Universidad Nacional Autónoma de México, Ciudad de Mexico 04510, Mexico; armandop@unam.mx (A.P.-T.); toriello@unam.mx (C.T.); 3 Centro de Ciencias Básicas, Universidad Autónoma de Aguascalientes, Av. Universidad No. 940, Aguascalientes 20100, Mexico; dcervantesga@conacyt.mx (D.C.-G.); jventur@correo.uaa.mx (J.V.-J.); 4 Consejo Nacional de Ciencia y Tecnología, Ciudad de México 03940, Mexico

**Keywords:** sporotrichosis, phagocytosis, immune response, virulence factors, dermal resident macrophages

## Abstract

The role of immune cells associated with sporotrichosis caused by *Sporothrix schenckii* is not yet fully clarified. Macrophages through pattern recognition receptors (PRRs) can recognize pathogen-associated molecular patterns (PAMPs) of *Sporothrix*, engulf it, activate respiratory burst, and secrete pro-inflammatory or anti-inflammatory biological mediators to control infection. It is important to consider that the characteristics associated with *S. schenckii* and/or the host may influence macrophage polarization (M1/M2), cell recruitment, and the type of immune response (1, 2, and 17). Currently, with the use of new monocyte-macrophage cell lines, it is possible to evaluate different host–pathogen interaction processes, which allows for the proposal of new mechanisms in human sporotrichosis. Therefore, in order to contribute to the understanding of these host–pathogen interactions, the aim of this review is to summarize and discuss the immune responses induced by macrophage-*S. schenckii* interactions, as well as the PRRs and PAMPs involved during the recognition of *S. schenckii* that favor the immune evasion by the fungus.

## 1. Introduction

Sporotrichosis is a subcutaneous mycosis frequently found in tropical and sub-tropical areas of Latin America and other countries throughout the world [1,2,3]. Its etiological agent, *Sporothrix schenckii*, was considered to be a unique species for almost a century. Currently, there are other *Sporothrix* species with significant importance in medical mycology, such as *S. brasiliensis*, *S. globosa*, *S. luriei*, *S. mexicana*, and *S. pallida*, all identified and classified by molecular biology techniques [4], mainly by those methods based on the detection of the calmodulin gene or the nuclear ribosomal internal transcribed spacer (ITS) region [5].

All species of *Sporothrix* are thermodimorphic fungi, presenting the filamentous saprophytic morphotype in soil, plants, and animal excreta or in vitro at 25 °C, and yeast parasitic morphotye in tissue host or in vitro at 35–37 °C. As in other subcutaneous mycoses, host infection occurs by traumatic inoculation through the skin of materials contaminated with fragments of hyphae or conidia of the fungus. Rarely, this infection is acquired by spore inhalation leading to a primary lung disease [4,6].

The fixed and lymphocutaneous lesions that involve skin and subcutaneous tissues are the most common clinical forms of sporotrichosis [7], and usually affect immunocompetent hosts. On the other hand, disseminated presentations, such as disseminated cutaneous, with or without visceral, osteoarticular, and pulmonary involvement, are more frequent in immunosuppressed patients [2]. In the majority of cases, treatment becomes imperative and, as an exception, spontaneous resolution occurs. The increased clinical severity is related to a decrease in the host’s immune and inflammatory responses, the clinical form of the disease, the species of *Sporothrix* involved, heavy fungal burden, and extensive dissemination [2,4,6,8].

The innate immune system is the host’s first line of defense against pathogens. Generally, this immune response is efficient and capable of controlling the infection without disease development, mainly in opportunistic infections. However, in immunocompromised hosts, pathogens can proliferate and establish the disease [9]. Surveillance and clearance of fungal pathogens are highly dependent on the phagocytic activity of macrophages and neutrophils [10,11]. The efficiency by which phagocytes recognize, internalize, and kill fungal pathogens depends on the size, shape, and composition of the fungal cells and the success or failure of various fungal immune evasion mechanisms [12]. Responses may also depend on the host’s species or model in which the macrophage–*S. schenckii* interactions are studied [13].

The fungal cell wall (CW) is the first point of contact between the host and the pathogen, consequently playing an important role in pathogenesis and immunogenicity, the latter considering that many CW components have been characterized as inducers of cellular and humoral immune response [14]. Changes in composition and/or structure in CW that occur over time in *Sporothrix* yeast cultures influence their recognition and phagocytosis by human monocyte-derived macrophages [15] or by murine macrophages [16], and cytokine secretion by human peripheral blood mononuclear cells (PBMCs) [17].

The key role of macrophages as innate immune response cells depends on the expression of surface receptors, which can be activated by factors derived from the host and pathogens [18]. A comprehensive analysis of the molecular factors of *S. schenckii* that activates receptors over the surface of macrophages has been performed by Alba-Fierro et al. [19]. This review tries to explain how the immune response induced by the fungus relies on the activated signaling pathway, the nature of the antigen, and the morphology of the fungus. These phagocytic cells are able to act as reservoirs for the immune evasion of intracellular pathogens and as triggers of specific immune responses mediated by antigen presentation and proinflammatory cytokines and chemokines secretion [20]. In this review, we will discuss the role of macrophages in the host’s defense against *S. schenckii* and the strategies used by the fungus for immune evasion.

## 2. Macrophages: A Brief Overview

Macrophages constitute a ubiquitous mononuclear phagocyte system (MPS) that is adaptable, regulated, and able to evoke defense responses, locally and systemically. The heterogeneous functions of macrophages are maintained through a fine regulation. To protect the host, the activities of macrophages (phagocytosis, oxidative burst, clearance, and cytokine secretion) contribute to the activation of innate and adaptive immune responses (afferent and efferent arms of the immune system, respectively) to control the infection, supporting the inflammatory reaction [21]. In a tissue injury, a similar process is activated but triggered by different molecular signals, allowing tissue repair [22].

Macrophages share the common origin from hemopoietic stem cells to monocytes, dendritic cells (DC), and osteoclasts [23,24,25]. However, they display considerable heterogeneity, differing widely in phenotype and morphological appearance, depending on their location [26,27]. Resident macrophages have a dual origin: (1) macrophages derived from erythromyeloid precursors (yolk sac and fetal liver precursors) and seeded throughout tissues, persisting in adults as resident self-maintaining populations, which perform phagocytic and organ-specific trophic functions [28,29]; and (2) bone marrow-derived blood monocytes that replenish resident macrophages, mainly following injury, infection, and sterile inflammation. Therefore, different tissues and host locations contain variable mixtures of embryonic origin and bone marrow-derived monocytes/macrophages. This dual origin determines the differences in the biosynthetic responses to endogenous and exogenous stimuli and a marked phenotypic heterogeneity [30].

Monocytes and macrophages express a wide range of molecules for the recognition and intake of self-derived, foreign particles and molecules by phagocytosis and endocytosis, respectively. Many of their secretory activities, including pro- and anti-inflammatory cytokines, proteases, chemokines, growth and differentiation factors, reactive oxygen species (ROS), and also reactive nitrogen species (RNS), are induced in response to microorganisms activating changes in gene expression [31]. In addition, macrophages are activated and differentiated by cytokines secreted from lymphocytes and other local tissue cells, and this activation gives them the capacity to respond to diverse challenges [32].

### 2.1. Macrophage Subtypes

Macrophages are involved in the homeostatic functions of organs, development, and tissue repair as well as in the defense against pathogens, chronic inflammation, fibrosis, and cancer [33]. Macrophages are not homogeneous, and their phenotypic heterogeneity correlates with unique functions and specificity to local microenvironments, enabling a plasticity to produce appropriate responses to pathogens or to signal molecules released by activated lymphocytes or damaged tissue. This heterogeneity is referred, mainly but not exclusively, to as polarization, which subdivides macrophages in terms of their activation as ‘classical’ (Th1/M1-type), or ‘alternative’ (Th2/M2-type) [21,34].

At the same time, subsets of blood monocytes in humans, mice, and other species with phenotypic differences and heterogeneity in their origin, maturation, and activation have been identified [27]. Human monocytes are divided into subsets based on surface CD14 and CD16 expression. CD14${}^{++}$CD16${}^{-}$, named classical monocytes, are the most prevalent monocyte subsets in human blood, and, similarly to mice monocytes Ly6C${}^{hi}$, these cells express the chemokine receptors CCR1 and CCR2. The CD16${}^{+}$ population is composed of two subsets, CD14${}^{+}$CD16${}^{++}$ and CD14${}^{++}$CD16${}^{+}$ monocytes (non-classical and intermediate monocytes, respectively). The CD14${}^{+}$CD16${}^{++}$ subset (patrolling) is similar to the mice Ly6C${}^{low}$ monocytes population [35,36,37].

#### 2.1.1. M1 Classical Monocytes (Inflammatory)

Classically activated M1 macrophages are responsible for the type 1 immune response, which is mainly formed by cytokines like interferon (IFN)-γ and tumor necrosis factor (TNF)-α, and induced by lipopolysaccharide (LPS)-bacterial related components and TLR analogs. These macrophages show higher antimicrobial capacity and are prototypical cells that produce pro-inflammatory mediators [38]; in addition, they are efficient in the generation of ROS, pro-inflammatory cytokines (interleukin (IL)-1β, TNF-α and IL-6), and cytotoxicity (phagocytosis of microorganisms and necrotic cells). These M1 monocytes are important in the innate immune protection against infectious pathogens. During an infectious challenge, M1 monocytes upregulate TNF-α, which activates inducible nitric oxide synthase (iNOS)-producing-DC known as TNF/iNOS-producing (Tip)-DC, which contributes to the development of adaptive immune responses. In mice, M1 macrophage-associated markers include IL-12, major histocompatibility complex (MHC) class II molecules, and iNOS [37,39].

#### 2.1.2. M2 Non-Classical Monocytes (Patrolling, Anti-Inflammatory)

Anti-inflammatory or alternatively activated M2 macrophages represent the promotion of type 2 immune response as its differentiation can be mediated by IL-4, mainly released from basophils and mast cells [32]. Significantly, these cells can produce IL-4 in response to chitin from fungi [40]. M2 macrophages have been shown to trigger the deposition of extracellular matrix proteins as arginase activity is induced by IL-4, and collagen production is facilitated by the conversion of arginine to ornithine [41]. It is known that signal molecules such as signal transducer and activator of transcription (Stat)1, Stat3, Stat6; suppressor of cytokine signaling (SOCS)-1; interferon regulatory factor (IRF)-4, as well as that a variety of miRNAs are involved in the macrophage polarization regulation. On the other hand, the expression levels or activity of the transforming growth factor (TGF)-β, IL-4, IL-10, and Stat3 are clearly elevated in M2 macrophages, so they are linked to anti-inflammatory actions and tissue repair [42,43]. There is evidence that these cells are derived from classical monocytes which can give rise to tissue-resident macrophages responsible for tissue healing promotion [44].

### 2.2. Macrophages Recruitment

During both homeostasis and inflammation, circulating monocytes leave the bloodstream and migrate into tissues where they are subdivided into subsets that differ in size, trafficking, innate immune receptor expression, and their ability to differentiate. Following stimulation with pro-inflammatory cytokines and/or microbial molecules or local growth factors, these cells differentiate into macrophage or DC populations [45]. Recruitment of monocytes is essential for an effective control and clearance of viral, bacterial, fungal, and protozoal infections, but recruited monocytes could also contribute to the pathogenesis of inflammatory and degenerative diseases [46].

### 2.3. Phagocytosis

The process of engulfment and destruction of invading microorganisms is called phagocytosis and is a critical part of the innate immune response. In addition, clearance of apoptotic bodies occurs through this process, an essential aspect of tissue homeostasis and remodeling [47]. Phagocytosis in macrophages is mediated by a broad range of receptors through their interactions with natural and altered self-components as well as by several microorganisms that trigger signaling events for actin-dependent particle internalization. Examples of receptors related to phagocytosis are class A (macrophage receptor with collagenous structure receptor—MARCO), B (CD36 receptor), D (CD68 receptor), scavenger receptor (SR) [48], mannose receptor (MR, CD206), Dectin-1 (CLEC7A) [49], complement-3 (CD11b/CD18) receptor, Fcγ receptors (FcγR), and DC-specific intercellular adhesion molecule-3-grabbing non-integrin (DC-SIGN, CD209) [47,50].

Receptor engagement around the entire particle appears to be required for the completion of the internalization process in all instances, culminating in the formation of a membrane-bound vesicle termed phagosome. Then, phagosomes need to be converted into a potent microbicidal organelle that is central for both innate and adaptive immunity. This process, termed phagosome maturation, occurs through the fusion of lysosomes, converting them into phagolysosomes that are more acidic (pH 5.5–6.0) and, thus, acquire microbicidal and degradative organelle properties. In this subcellular compartment, the digestion of proteins, lipids, and carbohydrates is mediated by the concerted action of proteases, lipases, nucleases, glycosidases, and phosphatases, mediating the complete disintegration of large complex structures, such as dead or dying microbes [47,50,51]. ROS production, during the respiratory burst of phagocytes, is important for the innate immune response. The enzyme complex nicotinamide adenine dinucleotide phosphate (NADPH) oxidase, which constitutes one of the most important sources of ROS, is responsible for the electron transfer from NADPH to O${}_{2}$ and the simultaneous production of the superoxide anion (O${}_{2}^{-}$). The superoxide dismutase can dismutate O${}_{2}^{-}$ to hydrogen peroxide (H${}_{2}$O${}_{2}$), and then other molecules can be produced such as hydroxyl radicals (OH${}^{-}$) and singlet oxygen (${}^{1}$O${}_{2}$). It is important to mention that myeloperoxidase, one of the main components of primary or azurophilic granules, can use H${}_{2}$O${}_{2}$ to oxidize halides into more toxic reactive compounds such as hypochlorous acid (OHCl) and chloramines [49].

Like ROS, RNS are important for pathogen eradication. The formation of NO in phagocytes is catalyzed by iNOS. However, RNS production is delayed because induction of iNOS expression requires phagocyte detection of pro-inflammatory cytokines like TNF-α [47].

## 3. *S. schenckii* Recognition by Macrophages

The establishment of an infectious disease is influenced by the immune status of the host, the pathogenicity of the fungus, as well as the established interactions between them. Although it is true that the mechanisms of infection, the site of infection, and the strain and burden of the pathogen, among other aspects, are relevant, a first step is the recognition of the infectious agent by the host to activate the innate immune response early, and then to link and activate a specific adaptative immune response, when applicable [52].

**Figure 1:**
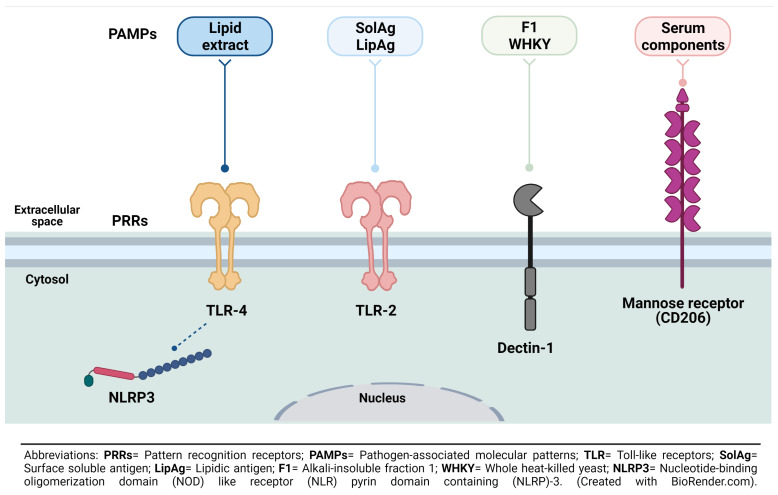
Receptors and ligands mediating *S. schenckii* recognition and phagocytosis by macrophages. Some PRRs like TLR-2 and -4, Dectin-1, and mannose receptor have been associated with recognition of some PAMPs as SolAg, LipAg, F1, WHKY, or some serum components associated with infection by *S. schenckii*. Thus far, it has only been proposed that the NLRP3 inflammasome can occur upon recognition of PAMPs by TLR-4.

Macrophages represent one of the most important lines of defense against a variety of pathogens, including *S. schenckii*. PRRs in macrophages such as TLRs, the nucleotide-binding oligomerization domain (NOD)-like receptors (NLRs) family pyrin domain containing 3 (NLRP3), and Dectin-1 have been studied in response to the recognition of PAMPs of this fungus, like cell surface antigens (either as hyphae or as yeast) and fungal cell surface lipids [53] (Figure 1). It is important to note that the differences in the CW architecture and composition of pathogenic fungi impact host recognition by innate immune cells [54].

TLRs are the best characterized PRR family because of the recognition of various fungal pathogens [55]. At present, more than ten TLRs are known, but only TLR-2 and TLR-4 have been involved in the immune response induced by sporotrichosis during the recognition of *S. schenckii* by macrophages and its inflammatory activation [56]. It is well known that TLR-2 recognizes fungal glycolipid phospholipomannan and zymosan, while TLR-4 recognizes the *O*-linked mannosyl residues, glucuronoxylomannan, and galactomannan [57].

The first PRR associated with *S. schenckii* recognition was TLR-4, and it has been involved in the secretion of pro-inflammatory and anti-inflammatory mediators. In peritoneal macrophages of mice with sporotrichosis, a correlation has been shown between the presence of TLR-4 and the secretion of both pro-inflammatory (TNF-α) and anti-inflammatory (IL-10) mediators during sporotrichosis. The nuclear translocation of the nuclear factor kappa-light-chain-enhancer of activated B cells (NF-κB) was elevated in the second week, coinciding with a significant increase in TNF-α during the first four weeks, which could suggest an inflammatory period of sporotrichosis, while higher levels of IL-10 production were observed in the final stages of the study, suggesting its possible role as an inhibitor of TNF-α secretion and, therefore, as an anti-inflammatory cytokine during infection. On the other hand, the secretion of NO, which increased between the 4th and 8th weeks, and the cellular apoptosis of peritoneal macrophages that was detected between the 4th and 6th weeks in mice may be involved in the immunosuppressive state observed at this time [58,59]. Something worth pointing out in this work is the fact that IL-1β, IL-6, and H${}_{2}$O${}_{2}$ increased their secretions during the entire ten weeks of infection and, although IL-1β decreased in the fourth week, it maintained higher concentrations as opposed to those in the non-inoculated control mice. In TLR-4-deficient mice, the TGF-β secretion increases, which could indicate that the secretion of this cytokine could negatively affect the inflammatory activation of macrophages during sporotrichosis in mice [60]. In in vitro assays, it was determined that TLR-4 played a less important role as opposed to TLR-2 during the interaction of *Sporothrix* with human PBMCs than with murine cells [17], which suggests interspecies differences that should be taken into account when explaining the immune response in this mycosis.

The role of TLR-2 in IL-1β, IL-12, IL-10, and TNF-α secretion and phagocytosis has also been demonstrated in mice’s peritoneal macrophages during a 10-week *S. schenckii* infection period. Remarkably, IL-12, as an important mediator for the activation of the type 1 response, together with NO, reached their maximum concentration at week 6 [53,61,62]. High levels of IL-12 are maintained by IFN-γ [63], which is a powerful activator of macrophages and type 1 cells that respond against fungal pathogens [64].

After TLR activation and the consequent production of pro-IL-1β and pro-IL-8 mediated by NF-κB, the assembly of the multimeric complex of NLRP3 forming the inflammasome promotes the proteolytic processing of pro-caspase-1 to active caspase-1, which cleaves pro-IL-1β and pro-IL-8 to produce mature secreting forms of IL-1β and IL-18, both important mediators of inflammation [65,66]. Inflammasome activation represents an important mechanism of protection against *S. schenckii* infection since macrophages from ASC${}^{-/-}$ and caspase-1${}^{-/-}$ null mice (both deficient in the activation of the inflammasome) have an impaired control of *S. schenckii* infection [67].

One more PRR associated with the recognition of *S. schenckii* is Dectin-1, which is a C-type lectin-like receptor and the major fungal β-1,3-glucans receptor on macrophages [68,69]. Like TLR-2 and TLR-4, Dectin-1 could stimulate peritoneal macrophages to secrete pro-(TNF-α and IL-1β) and anti-inflammatory (IL-10) mediators, increasing either its phagocytic activity or NO release [70]; in addition, this receptor has an important role in cytokines secretion against yeast and conidia of *S. schenckii*, mainly IL-10 in human PMBCs [17].

## 4. Macrophages and the Immune Responses in Sporotrichosis

The principal components of innate immunity are the skin and mucous membranes, which contain antigen presenting-dendritic cells and other migratory phagocytic cells, expressing constitutively PRRs that recognize PAMPs and secrete soluble mediators, generating inflammation reactions and supporting the development of the adaptive immune responses. Cellular and soluble components of innate immunity respond immediately in a coordinated manner after antigen recognition [71,72]. These essential functions of innate immunity for controlling the growth of *S. schenckii* yeast cells were revealed in mice with a defective mechanism to generate ROS—a lack of the NADPH oxidase function—whereby defective mice developed disseminated lethal sporotrichosis after subcutaneous inoculation of the fungus, in contrast to wild-type mice who controlled the infection and survived [73]. Since then, several studies have demonstrated the role of CD4${}^{+}$ T cells and activated macrophages during sporotrichosis infection and pathogen clearance in athymic nude [74,75,76,77] and Swiss mice [78,79]. These findings pointed to macrophages being relevant cells in the afferent and the efferent arms of the immune response against *S. schenckii*, in the context of type 1 and type 17 protective responses [80,81,82]. Infected and inflamed tissues release damage-associated molecular patterns (DAMPs) also recognized by PRRs. Macrophages participate in the structural remodeling after tissue damage as anti-inflammatory cells (M2 macrophages), in the setting of type 2 responses. In an ex vivo sporotrichosis model, after *S. schenckii* infection, using peritoneal exudate cells challenged with CW peptide-polysaccharide, macrophages M2 were the predominant cell population. These alternative activated macrophages expressed peaks of arginase-I activity as well as IL-10 and TGF-β production during the 6th and 8th weeks after infection [83]. The timing of macrophages participation in *S. schenckii* infection or in sporotrichosis should be considered in in vivo studies, particularly in order to understand the immunopathogenesis of sporotrichosis in humans. However, most of the *S. schenckii*-infected humans do not develop sporotrichosis, and rarely do they develop the systemic forms of the disease [84]. Murine models and in vitro studies have provided a wealth of information on the pathogenicity and virulence mechanisms of the fungus and on the cellular and molecular responses by the host’s immune system. Extrapolation of these results to explain the immunopathogenesis of sporotrichosis in humans should be done cautiously. An example of the latter are the studies related to the involvement of macrophages in the immunopathology of this disease [85]. It is common to overlook the morphological and functional heterogeneity of these innate immune cells and the relevance of tissue microenvironments in which the macrophage-*S. schenckii* interaction naturally occurs. Temporal and functional plasticity is a distinctive feature of macrophages as they act as host cells, effector cells, immunoregulatory cells, and tissue repair cells [30]. In this respect, it is pertinent to ask whether dermal resident macrophages, peritoneal macrophages, inflammatory monocytes, and the human monocytic cell line THP-1 are equivalent for studying the infection’s timing with a dimorphic fungus such as *S. schenckii*.

**Figure 2:**
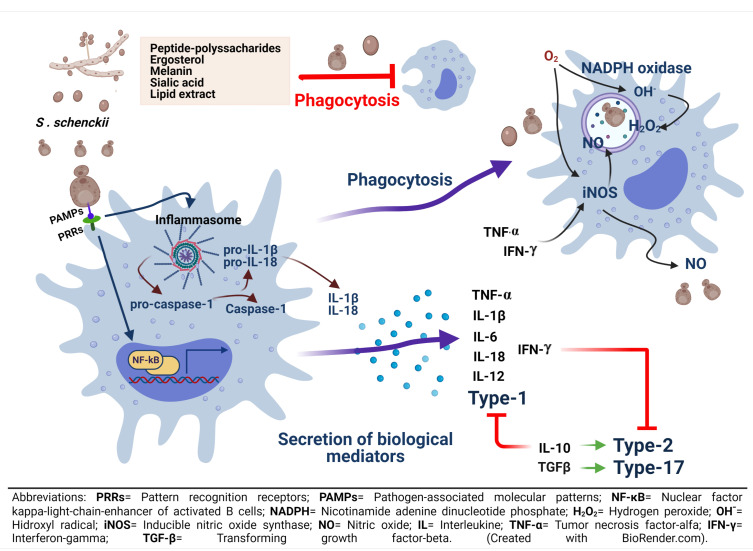
Recognition of *S. schenckii* and macrophages activation. After recognition of cellular PAMPs of *S. schenckii* by macrophage PRRs, phagocytosis and secretion of biological mediators can be activated. Phagocytosis and respiratory burst, with the participation of NADPH oxidase and the consequent formation of ROS, such as H${}_{2}$O${}_{2}$, will focus on fungal clearance within the phagosome. On the other hand, the formation of RNS, such as NO, with the participation of iNOS, should have the same function as ROS; however, NO has been associated with a state of immunosuppression after several weeks of infection. It is also important to note that cellular components of *S. schenckii* can prevent its phagocytosis by macrophages. In addition to phagocytosis, secretion of biological mediators associated with nuclear translocation of NF-κB can occur. This translocation, with the participation of caspase-1, can promote the assembly of the NLRP3 inflammasome, inducing cytokine secretion. In any case, the secretion of biological mediators can promote either inflammatory (TNF-α, IL-1β, IL-6, IL-12, IL-18, IFN-γ) (cellular response type 1) or anti-inflammatory (IL10, TGF-β) (cellular response type 2 and 17) processes. Interestingly, TNF-α and IFN-γ) may be related with the activation of iNOS and the formation of NO. Furthermore, IFN-γ can also block type-2 and 17 responses, while IL-10 can block type 1 response.

The ingestion of fungal cells of *S. schenckii* triggers the oxidative burst in murine macrophages, and their stimulation with IFN-γ induced NO production and inhibition of fungal growth, indicating that NO is a fungicidal mediator against *S. schenckii* in vitro [86]. However, this fungicidal activity and NO production by IFN-γ and LPS-activated macrophages were abolished after the infection of yeast cells in a murine model of systemic sporotrichosis. Moreover, mice deficient in inducible nitric oxide synthase (iNOS${}^{-/-}$) and C57BL/6 wild-type (WT) mice treated with N-omega-nitro-arginine, an iNOS inhibitor, presented fungal resistance, controlling fungal load in tissues, and restoring T-cell activity, as well as producing high amounts of IFN-γ. These findings suggest that the activation of the NO system in vivo contributes to the immunosuppression and cytokine balance during the early phases of infection with *S. schenckii* [87]. In human sporotrichosis, activation of the NO system or the iNOS expression has been correlated with the intensity of the inflammatory infiltrate and the number of neutrophils in chronic skin lesions from the lymphocutaneous form, compared to the fixed form, observing a greater infiltrate in the lymphocutaneous form, a more common clinical presentation of sporotrichosis [88]. In addition, the lymphocutaneous form presented a higher percentage of CD4${}^{+}$ T cells, and CD22${}^{+}$ B cells, than the fixed form, but no differences in macrophages, CD3${}^{+}$, and CD8${}^{+}$ cells were observed in the two disease forms. Despite the higher expression of iNOS and the lack of differences in macrophages, a great fungal burden was found in skin lesions from the lymphocutaneous form, compared to the fixed form. Thus, this ineffective fungus control in the lymphocutaneous form could be related to a higher virulence of some *S. schenckii* strains as demonstrated in animal models [89,90]. Recently, it has been proposed that the immunopathological and clinical characteristics of the lymphocutaneous form of sporotrichosis may indicate either an initial uncontrolled or an ineffective inflammatory process that characterizes an unbalanced immune response [91]. Fixed lesions commonly present more focal inflammatory infiltrates, surrounded by fibrous tissue that could impair dissemination of the fungus, facilitating its control. Probably, alternative activated macrophages with arginase-I activity as well as with IL-10 and TGF-β attenuate the inflammatory response and activate fibroblasts for collagen synthesis via TGF-β [92]. On the other hand, the presence of intense and diffuse, non-fibrotic inflammatory infiltrate, with intense necrosis and suppurative reactions, could facilitate lymphatic dissemination of the fungus in the lymphocutaneous form of human sporotrichosis [93]. Since both clinical forms showed no differences in the percentage of macrophages, but rather in the percentage of neutrophils, which was higher in the lymphocutaneous form [88], it may be suggested that, in the fixed form, the less severe form of sporotrichosis, an efficient collaboration between macrophages and neutrophils occurs to limit and repair tissue damage, decreasing the fungal burden and preventing the spread of the infection. The immune response mechanism involved in the macrophage-*S. schenckii* interaction is summarized in (Figure 2).

## 5. Macrophage-*S. schenckii* Interaction: Some New Aspects

Optimal binding and phagocytosis of *S. schenckii* conidia by the human monocytic cell line THP-1 require opsonization with normal human serum components, which are recognized by MR. Opsonized conidia uptake stimulates the production of ROS, resulting in the killing of conidia. Interestingly, THP-1 cells appeared to use complement receptors to phagocytize yeast cells, followed by ROS production. Release of TNF-α was not stimulated by opsonized or non-opsonized conidia, whereas opsonized and non-opsonized yeast cells did release it. The different pro-inflammatory response induced by *S. schenckii* morphotypes could be related to the progression of the inflammation after the natural infection with this fungus [94]. Some of these results were confirmed recently, using human monocyte-derived macrophages incubated with non-opsonized or opsonized conidia with 10% normal human serum or serum proteins absorbed into conidia as albumin, transferrin, serum amyloid P component (SAP) or α1-antitrypsin (AAT) identified by tandem mass spectrometry. In addition, it was demonstrated that conidia phagocytosis depended on the concentration of SAP or AAT, and it is worth pointing out that the competition assay with D-mannose did not affect macrophage phagocytosis, which suggests that the MR is not involved. Transferrin or albumin did not have any effect on conidia uptake. This work opens up new routes in investigating the role of other innate immunity proteins in fungal diseases and in systemic or deep sporotrichosis [95]. The SAP is a hepatocyte secreted circulating lectin with specificity for the cyclic 4,6-pyruvate acetal of galactose [96], which can bind to the surface of some bacteria and fungi, helping the complement-mediated immunity [97]; in addition, this lectin can be identified as a friend or foe of the host in bacterial and fungal infections [98,99,100]. Macrophage phagocytosis and the concomitant reduction of inflammatory cytokines have been observed after SAP binds to functional and pathogenic amyloid on the surface of fungi such as *Candida albicans* [101,102,103], *Aspergillus*, Mucorales, and *Coccidioides* [100] that produce deep mycoses.

On the other hand, AAT, one of the most abundant serine protease inhibitors, has anti-inflammatory activity against key innate immune response cells such as neutrophils, macrophages, monocytes, and mast cells [104], and it could be relevant to explore them in natural and experimental *S. schenckii* infections and in the most common clinical forms of sporotrichosis [88]. The rapid recruitment of neutrophils to injury or infection sites is mediated by IL-8, also known as CXCL8 (a chemokine produced by macrophages and endothelial cells). Such recruitment is a hallmark of the inflammatory response and is required for the host’s effective defense against pathogenic stimuli [105]. However, neutrophils can also lead to chronic tissue destruction, facilitating, as it has been mentioned previously, the lymphatic dissemination of *S. schenckii* in the lymphocutaneous form of human sporotrichosis and altering the antifungal cooperation with macrophages [88]. The ATT, as a regulatory enzyme that inhibits neutrophil proteases’ activity, such as elastase [106], would limit host–tissue injury, attenuating the inflammatory response and avoiding the fungus dissemination, as in other infections.

Hepatocytes, the major parenchymal cells in the liver, produce SAP and ATT, and hundreds of other acute-phase proteins, exhibiting a wide variety of functions including the activation of innate immunity [107]. It is well known that customary consumption of alcohol can cause chronic liver damage and hepatic dysfunction. It is likely to assume that the altered secretion of SAP and ATT, along with other immunosuppressive mechanisms present in alcoholic patients, contributes to the development of disseminated cutaneous and pulmonary sporotrichosis [2,108,109,110]. However, liver resident macrophages could be also implicated.

Hepatic macrophages, also known as Kupffer cells, are F4/80${}^{+}$ phagocytes that downregulate CR3 but express CRIg (a tissue-specific complement receptor) and CLEC4F (a liver-specific C-type lectin for alpha-galactosyl ceramide), which defines their innate recognition function and adhesion [111]. Kupffer cells also express the MR, involved in clearance of mannosylated glycoconjugated [112], and the class A scavenger receptor (SR-A), a multiligand and multifunctional receptor of polyanionic ligands [113]. The interactions between the Kupffer cells and fungal pathogens are scarcely understood, and *S. schenckii* is not the exception. The disseminated sporotrichosis models in mice with liver involvement have set aside the study of the Kupffer cells, the body’s largest population of resident macrophages. As embryonic-derived macrophages, Kupffer cells induce liver metabolic responses such as biosynthesis of acute phase plasma proteins to combat acute and chronic infections [114]. It is likely that cytokines mediate interactions between hepatocytes and Kupffer cells during fungal infections. Experiments with a primary culture of murine TLR-4-deficient Kupffer cells from C57BL/ScCr mice suggest that the cytokine response (TNF-α, chemokines keratinocyte-derived chemokine (KC) and MIP-2) to the fungal component of *A. fumigatus* and *C. albicans* hyphae and conidia is not mediated by TLR-4 but by tyrosine kinases [115]. This contrasts with the results obtained using peritoneal macrophages from TLR-4-deficient (C3H/HeJ) and control mice (C3H/HePas) infected with *S. schenckii* yeast cells, where significantly greater amounts of pro-inflammatory mediators, such as NO and TNF-α (early-stage post-infection), and anti-inflammatory cytokines, such as IL-10 (late-stage post-infection), were produced by thioglycollate-elicited peritoneal macrophages from infected C3H/HePas mice [58]. In this work, the authors suggest that other receptors like TLR-2 and Dectin-1 may also contribute to the immune response during *S. schenckii* infection, but they assign TLR-4 an important role in governing the functions of macrophages in fungal infection. However, the origin and functional status of resident macrophages used in similar studies must also be considered.

Much of the knowledge about the macrophage-*S. schenckii* interaction derives from in vitro and in vivo studies using murine peritoneal macrophages which, as Kupffer cells, are F4/80${}^{+}$. However, the origin (ontogeny), differentiation, phenotypic differences, and the organ-specific trophic functions of macrophages are heterogeneous variables that could mainly limit an interspecies extrapolation of conclusions, and, moreover, in terms of a human perspective [26]. Furthermore, the peritoneal cavity is not the most frequent site of entry for *S. schenckii*. This serosa contains an independent reserve subpopulation of large and mature resident macrophages that expresses the transcription factor GATA-6. They migrate rapidly to regional lymph nodes after stimulation and are recruited after sterile injury to the liver, acquiring an alternative activation phenotype (M2) characterized by anti-inflammatory and tissue damage repairing actions [43,116,117]. Thus, what kind of macrophage peritoneal subpopulation is suitable to study the infection by the *Sporothrix* species?

Phagocytic capacity is variable among resident macrophages from different organs and is mediated by distinct repertoires of receptors, opsonins, and transcription factors from each tissue, indicating that heterogeneity is established by local tissue-derived factors [118]. The liver and the peritoneal cavity exemplify this fact, increasing the complexity by containing several macrophages of distinct origins, whose activation and function most likely reflects a spectrum of changes, rather than the simplistic M1/M2 binary division [32]. This concept can be extended to the skin, the host’s natural way by which more frequently *Sporothrix* species enter.

### A New Population of MR${}^{hi}$/Arg-1 and iNOS Negative Dermal Resident Macrophages in Mice

The ontogeny and tissue-derived signals shape the functional specialization and plasticity of macrophages [119,120]. The origin of macrophages plays an important role in their functional adaptation in accordance with the limited reprogramming of peritoneum-resident macrophages, compared to recruited monocyte-derived macrophages in an infection-driven inflammatory model [121].

Little is known regarding the plasticity of dermal resident macrophages and their relative contributions to antimicrobial immunity or to pathology in cutaneous infection. Under steady-state conditions, mice’s dermis contains a population of M2-like resident macrophages of embryonic origin, so they are not replaced by recruited blood precursors (monocytes or other bone-marrow derived cells). During infection by the highly virulent *Leishmania major* Seidman, these dermal macrophages promote an MR–dependent fashion and non healing cutaneous disease, similar to inflammatory response accompanied with CD4${}^{+}$ cell in lymphocutaneous sporotrichosis [91]. These MR high (MR${}^{hi}$) (or CD206${}^{+high}$) dermal resident macrophages, including CD36${}^{-}$, CD209${}^{-}$, and CD301 (macrophage galactose-type calcium-type lectin, MGL or CLEC10A)-positive, are locally maintained by eosinophil-derived IL-4 and IL-10 and retain M2 functionality despite the high levels of IFN-γ and TNF-α produced by activated monocyte-derived subsets within the same tissue environment [122,123]. Notably, these embryonic M2-like dermal resident macrophages were negative for arginase-1 (Arg-1) as well as iNOS, and their gene expression profiles are likely to be distinct from other M2 populations promoted by a specific set of cytokines and microbial stimuli in vivo or in vitro [124]. The CD206${}^{high}$ M2-like dermal macrophages share some similarities with pleural cavity resident macrophages, whose renewal after a helminth infection in the pleural cavity (polarized type 2 response) resulted in IL-4–dependent proliferative expansion with minimal recruitment of adult bone marrow–derived cells [125]. Interestingly, self-renewal and functional attributes of M2-like dermal macrophage cells occurred within a strong proinflammatory environment of the *L. major*-loaded dermis.

The CD206${}^{high}$/M2-like dermal resident macrophages could play an important role in all evolution stages of experimental skin infection with *S. schenckii* because the MR has already been involved in the binding and phagocytosis of opsonized conidia [86], without the release of TNF-α, while conversely releasing TNF-α and producing ROS induced by phagocytosis of opsonized and non-opsonized yeast. Dermal macrophages may have lost their responsiveness to TNF, which would normally function to antagonize their alternative activation [126]. This suggests that the dermis would have a proclivity to mount M2 macrophage-mediated responses in murine models of infection from the beginning of the interaction with some pathogens. This early response would be added to the alternative activation of M2 macrophages recruited and derived from monocytes after several weeks of infection development. The latter agrees with the results obtained after intraperitoneal inoculation of mice with yeast cells of *S. schenckii*. A predominance of M1 macrophages was demonstrated during the 2nd and 4th weeks post-infection, but, during the 6th and 8th weeks after infection, a predominance of CD206${}^{+}$ M2 macrophages, in response to type 2 cytokines, was identified [83]. The population of MR${}^{hi}$ M2 dermal macrophages was identified by combining MR and Ly6C staining on CD11b${}^{+}$lin${}^{-}$ cells. Their functional specialization for apoptotic cell capture and the local type 2 cytokines contribute to establishing and maintaining their M2 activation program [91].

## 6. The Macrophage-*S. schenckii* Interaction: Virulence/Pathogenicity or Survival Factor?

Macrophages play a key role in the host’s response against pathogens. The expression of cell surface receptors activated by molecules derived from both the host and pathogens are relevant to the macrophage’s innate immune response [18]. However, these phagocytic cells act either as hosts/niches for intracellular pathogens evading the immune responses or as antigen-presenting cells and promoters of an adaptive immune response [20]. Tachibana et al. [77] have demonstrated the role of macrophages against *S. schenckii*. These authors used previously subcutaneously immunized mice to obtain lymph node cells which were adoptively transferred to naive congenitally athymic nude mice. The ability to transfer protection against a *S. schenckii* infection was significantly reduced when lymph node cells were depleted of CD4${}^{+}$ T cells and abolished when macrophages of mice were blocked with carrageenan. An in vitro study, using either immune lymph node cells alone or macrophages alone, failed to kill the fungus. However, inhibition of fungal growth was observed when both immune lymph node cells and macrophages were combined [77].

Macrophage receptors play an important role in the development of an effective innate immune response against pathogens since they mediate phagocytosis, signaling cascades, intracellular traffic, inflammatory response, and antigen presentation [48]. The receptors involved during *S. schenckii*’s antigen recognition as well as the activated signaling cascades have been previously reviewed [19]. Although Martínez-Alvarez et al. [17] observed variations in the chitin content of the CW of conidia, yeasts, and germ tubes of *S. schenckii*, the proportion of carbohydrates (rhamnose and glucose) is similar in all of them. This could explain why they are all capable of activating the secretion of pro-inflammatory cytokines from PBMCs. On the other hand, Lopes-Bezerra et al. [15] showed that 4-day-old *S. schenckii* yeasts were more easily engulfed than 10-day-old ones by human monocytes. Additionally, they observed that the fungus shed layers of the CW during its growth, which can cause antigenemia or inflammation at a distance from the site of the pathogen stem cell. In addition, if shed layers of CW contain PAMPs, these can function as decoys for PRRs in macrophages and evade phagocytosis, which could influence the infection process and disease development. Phagocytosis of serum opsonized *S. schenckii* conidia by the human monocytic cell line THP-1 requires the recognition by MR, and it induces the production of ROS, resulting in the killing of conidia. This monocytic cell line produces ROS after phagocytose yeast cells using complement receptors. Release of TNF-α was not stimulated by opsonized or non-opsonized conidia, whereas opsonized and non-opsonized yeast cells did release it. These findings highlight the relevance of *S. schenckii* morphotypes to induce different pro-inflammatory responses that could be related to the progression of the natural infection with this fungus [94], and they suggest that dimorphism is a pathogenicity factor more than a survival factor for *S. schenckii* [127,128,129,130,131]. However, neither *S. schenckii* morphology nor macrophages receptors are the only features that influence the macrophage-*S. schenckii* interaction since the composition of the surface of the fungus plays a lead role. In this regard, Sgarbi et al. [132] studied the presence of ergosterol, a compound on the surface of *S. schenckii* capable of sequestering ROS and avoiding the damage produced by effector cells from the immune response such as macrophages. Remarkably, this study demonstrated that an enzymatic extract obtained from the same fungus reverted ergosterol peroxide to ergosterol, regenerating ergosterol and increasing the resistance of the fungus to ROS, which enhances the virulence of the fungus. Ergosterol has been also associated with antifungal resistance. Brilhante et al. [133] showed how diminishing the concentration of ergosterol through terpinene-4-ol, a monoterpene that exhibits antifungal activities increases the susceptibility of *S. schenckii* to itraconazole, amphotericin B, and terbinafine, the first choice antifungals used for sporotrichosis treatment. Furthermore, Borba et al. [134], using an inhibitor for Δ(24)-esterol methyltransferase, an enzyme that participates in ergosterol biosynthesis, increased the susceptibility of *S. schenckii* to itraconazole, suggesting a new role of ergosterol in the virulence of the fungus. According to the latter works, it must be noted that a diminishment in ergosterol levels interferes with the structure of *S. schenckii*’s CW, a structure that mediates its interaction with immune response cells such as macrophages.

Antifungal resistance of *S. schenckii* to macrophages has also been associated with melanin production [135]. Almeida et al. [136], using inhibitors for eumelanin and pyomelanin production during *S. schenckii* growth in the presence of terbinafine, a recommended therapeutic alternative for sporotrichosis treatment, demonstrated that melanin content has the potential to protect *S. schenckii* and *S. brasiliensis* strains from antifungal effects induced by terbinafine since the presence of melanin increased the minimal inhibitory and fungicidal concentrations. However, the potential protector role of melanin on different species of the *Sporothrix* complex such as *S. globosa*, *S. luriei*, and *S. pallida* has been and remains poorly studied. In vitro studies have also shown that *S. schenckii* conidia without melanin production are highly phagocyted, compared with melanin pigmented conidia, which indicates that melanin contributes to fungal survival within the host, increasing its virulence [137]. Fungal cells’ melanization can also affect the pathogenesis of the fungus since pigmented isolates of *S. schenckii* have demonstrated a higher ability to invade tissues, compared with albino strains [138].

Yeast morphotype of *S. schenckii*, contrary to the conidial phase, possesses two concanavalin A (Con A) reactive layers on the external surface of the CW. These layers can break off from the cell during its interaction with immune response cells. This released material from *S. schenckii*’s CW contains peptidorhamnomannan and galactomannan antigens, as well as anionic surface groups detectable through reactions with ferritin. These polysaccharide antigens purified from the CW of *S. schenckii* inhibited in vitro yeast cell phagocytosis by murine peritoneal macrophages [139]. Conversely, the treatment of yeast cells of *S. schenckii* with neuraminidase diminished the presence of sialic acid and modified the structure of both surface layers, increasing the phagocytosis index of the fungus [140].

Galactomannans and rhamnomannan polysaccharides have been suggested as regulators or inhibitors of the immune response since macrophages incubated with these compounds increase the production of L-arginase, TGF-β, and IL-10, molecules with anti-inflammatory activities. In fact, peptide-polysaccharide antigens expressed at the CW of yeast cells of *S. schenckii* could be dual activators of macrophages, capable of inducing the classical pathway (M1) and a type 1 response, as well as of eliciting the alternative pathway (M2) and a type 2 response, according to the fungal infection course [83], and they can also diminish cellular response in murine sporotrichosis models [79]. The inhibition of the in vitro phagocytosis, along with the induction of a high release of TNF-α and NO, has also been attributed to a lipid component from the CW of *S. schenckii* [141]. Moreover, soluble and lipidic antigens from the fungal cell surface can activate macrophages by targeting their TLR-2, increasing the secretion of TNF-α, IL-1β, and IL-10 [53], whereas the same lipidic antigen increases the production of TGF-β in macrophages isolated from TLR-4 deficient mice [58]. Additionally, it has been demonstrated that murine splenic macrophages increase the production of IFN-γ and NO after being stimulated with an exoantigen of *S. schenckii*; however, peritoneal macrophages could be induced to express early classical activation, during the first few weeks, and then an alternative activation in the late stages of a systemic infection of Swiss mice with *S. schenckii* yeast cells [63,82]. Lipidic antigen activates caspase-1 [142], which plays a fundamental role in innate immunity and in several important inflammatory diseases. This protease activates the pro-inflammatory cytokines proIL-1β and pro-IL-18, which could induce pyroptosis, a caspase-1-dependent type of programmed cell death. In addition, there is evidence that caspase-1 supports cell survival by activation of NF-κB, induction of membrane repair, and regulation of unconventional secretion of certain proteins [65]. Some of these partially opposing effects of caspase-1 activation by a lipidic antigen from the CW of *S. schenckii* have not been studied neither in human sporotrichosis nor in murine models of this fungal disease. Understanding the virulence/pathogenicity of *S. schenckii* should allow for the consideration that different subsets of macrophages, from different microenvironments and mammalian species, have a different potentiality to recognize, phagocytose, and eliminate both conidia and yeast cells of *S. schenckii*; in addition, it should allow for bearing in mind that this fungus is able to modulate the macrophage’s activation, inhibiting or increasing the production of ROS and RNS that does or does not enhance the overall defense immune responses of the host. In the natural and in vivo experimental infections, other innate immune response cells such as DC, mast cells, and neutrophils participate with other defense mechanisms to give a more comprehensive and integrated response by the host to control the infection by *S. schenckii*. Finally, conidia, mycelia, and the yeast forms of *S. schenckii* secrete active enzymes as acid phosphatase that participate as a virulence factor during the macrophage-fungus interaction [143,144].

## 7. Conclusions

In the immune system, human cells participate against microbial pathogens. Macrophages play an important role in both the activation of the innate immune system and the elimination of the pathogenic forms of the genus *Sporothrix*. Macrophage receptors play a key role in activating an effective immune response against the fungus, as they mediate phagocytosis, signaling cascades, intracellular trafficking, inflammatory responses, and antigen presentation. Macrophages can be activated by factors derived from the host and the pathogen. Macrophage PRRs such as TLRs, NLRs, MR, and Dectin-1 have been studied in response to recognition of PAMPs in *S. schenckii*. This fungus has different pathogenicity/virulence factors, such as ergosterol, melanin, polysaccharide peptides, and lipid components, some of them located on the fungal cell surface, which are involved in macrophage evasion mechanisms (Figure 2). Furthermore, before extrapolating from in vitro and in vivo studies, it is convenient to consider the morphological and functional heterogeneity of these phagocytic cells and the tissue microenvironments in which the macrophage-*S. schenckii* interaction occurs, to explain the immunopathogenesis of sporotrichosis in humans. Likewise, it is necessary to know even more about the immune response induced during the mycelial morphotype infection, which represents the natural infection process.

## Data Availability

Not applicable.
